# Baseline BMI is associated with clinical symptom improvements in first-episode schizophrenia: a longitudinal study

**DOI:** 10.3389/fphar.2023.1264591

**Published:** 2023-11-09

**Authors:** Xiaofang Chen, Yong Fan, Wenchao Ren, Maodi Sun, Xiaoni Guan, Meihong Xiu, Shuyun Li

**Affiliations:** ^1^ Beijing Huilongguan Hospital, Peking University Huilongguan Clinical Medical School, Beijing, China; ^2^ Qingdao Mental Health Center, Qingdao, China; ^3^ North University of China, Taiyuan, China; ^4^ Department of Nutritional and Metabolic Psychiatry, The Affiliated Brain Hospital of Guangzhou Medical University, Guangzhou, China; ^5^ Guangdong Engineering Technology Research Center for Translational Medicine of Mental Disorders, Guangzhou, China; ^6^ Key Laboratory of Neurogenetics and Channelopathies of Guangdong Province and the Ministry of Education of China, Guangzhou Medical University, Guangzhou, China

**Keywords:** schizophrenia, weight, improvements, negative symptoms, risperidone

## Abstract

**Background:** There is sufficient evidence of the high prevalence of obesity in schizophrenia (SZ) compared to the general population. Previous studies have reported that weight gain correlated with the response to antipsychotics in patients with SZ. Nonetheless, the relationship between body mass index (BMI) and therapeutic benefits remains unclear. This study was designed to investigate the association between baseline BMI and improvements in clinical symptoms after treatment with antipsychotics in first-episode and medication-naïve SZ (FEMNS).

**Methods:** A total of 241 FEMNS patients were enrolled and received risperidone over 12 weeks. The severity of symptoms was assessed by the Positive and Negative Syndrome Scale (PANSS) and BMI was measured at baseline and 12-week follow-up.

**Results:** We found that risperidone treatment raised the body weight of FEMNS patients and baseline BMI was negatively correlated with the improvement in negative symptoms (r = −0.14, *p* = 0.03) after 12-week treatment. Linear regression analysis indicated that baseline BMI was an independent predictor of response to risperidone in the early stage of SZ.

**Conclusion:** The current study suggests a close relationship between baseline BMI and improvement in negative symptoms in SZ.

## 1 Introduction

Atypical antipsychotics are reported to be associated with severe side effects like weight gain, obesity, and metabolic dysfunction (Li et al., 2018; Luckhoff et al., 2019; Li et al., 2021). It is reported that the prevalence of obesity ranges from 10% to 60% in SZ patients (Naslund et al., 2016; Annamalai et al., 2017). Weight gain/obesity impacts the quality of life and adherence to antipsychotic drugs in SZ (Dibonaventura et al., 2012; Pillinger et al., 2020).

A large of evidence has identified weight gain as a prognosis biomarker of the response to antipsychotic drugs (Ascher-Svanum et al., 2005; Bai et al., 2006; Sharma et al., 2014), underscoring the need for early monitoring of weight in SZ patients. Even in adolescents with SZ, substantial decreases in global psychopathology have been reported to be associated with weight gain after taking antipsychotic medications (Sharma et al., 2014). Studies have demonstrated that risperidone was associated with intermediate weight gain (1.76 kg), compared with olanzapine with the highest (3.45 kg) (De Hert et al., 2011). It is reported that individuals who were younger, female and first-episode and were less exposed to antipsychotics previously were more likely to increase the risk of obesity.

Antipsychotics have different treatment outcomes for patients with SZ (Zhu et al., 2022). Some studies have revealed the prognostic role of weight gain after treatment with antipsychotics in favorable treatment outcomes (Chen et al., 2021). However, considering the independence from confounding factors, such as treatment adherence and duration, the clinical significance of such a relationship has been questioned (Correll et al., 2011; De Hert et al., 2011; Hermes et al., 2011). Notably, a previous study has reported that baseline BMI correlated with antipsychotic-induced weight gain, and it was a predictive biomarker in the therapeutic response to antipsychotics (Verma et al., 2009). Another study in adolescents with SZ revealed that olanzapine-associated weight gain was not independently correlated with therapeutic response to olanzapine, but baseline obesity was related to more olanzapine-associated weight gain and symptomatic outcome (Kemp et al., 2013).

Studies have shown that the first-episode and medication-naive SZ (FEMNS) patients display the advantage of reducing the possible impacts of the use of antipsychotics, the duration of illness, and the impact of comorbidities associated with chronic illness (Xiu et al., 2020). In exploring the link between BMI and response to antipsychotics, thus, we recruited FEMNS patients in the current study. This study was designed to determine the predictive role of baseline BMI in the symptom improvements after 12-week treatment with risperidone in SZ.

## 2 Materials and methods

### 2.1 Patients

FEMNS patients were recruited from Beijing Hui-long-guan Hospital and Zhu-ma-dian Hospital. Patients were diagnosed with SZ as the Structured Clinical Interview for DSM-IV (SCID). The inclusion criteria were: 1) male and female inpatients; 2) first onset of psychosis; 3) between the ages of 18 and 45; 4) duration of illness less than 5 years; 5) previous antipsychotics exposure less than 14 days; 6) without substance abuse except for tobacco; 7) without major medical illness, such as diabetes, metabolic syndrome, hypertension, and cardiovascular disease; and 8) without taking weight-loss drug.

FEMNS patients received a flexible dose of risperidone for 12 weeks. During this 12-week study, all participants were hospitalized and nurses monitored compliance with risperidone. The protocol was approved by the ethical committee of Beijing Hui-long-guan Hospital (Ethic No.: 2011-4). Written informed consent was obtained from each patient.

### 2.2 BMI measurement

Height was determined by a metric stadiometer after removing the shoes. Weight was determined following overnight fasting at baseline and 12-week follow-up. Weight was measured in a hospital uniform with the pocket empty and without shoes. BMI was calculated using the weight divided by the height. All measurements were taken twice for each patient, and the mean was recorded.

In the present study, patients were classified into a high-BMI group when their BMI was 24 kg/m^2^ or higher and a low BMI group when their BMI was lower than 24 kg/m^2^, as in previous studies (An et al., 2018; Chen et al., 2023).

### 2.3 Clinical symptoms

The severity of symptoms was evaluated using the Positive and Negative Syndrome Scale (PANSS). The interviewers were trained before the assessment. After training, the inter-observer correlation coefficient for the PANSS total score was maintained at >0.8 during repeated assessments. PANSS scales were assessed at baseline and the end of 12 weeks. Improvement in clinical symptoms was calculated as the changes in PANSS score between baseline and 12-week follow-up after treatment with risperidone.

### 2.4 Statistical analysis

All statistical analyses were conducted using SPSS version 20.0. Statistical significance was defined as *p* < 0.05.

As described previously, the Last-observation-carried-forward (LOCF) was used for the data of the last time point of patients who dropped out (Wang et al., 2014; Raven et al., 2020; Wimms et al., 2020). ANOVA and *X* ^2^ test were used to investigate whether there was a difference in demographic and clinical characteristics, body weight and BMI between the groups at baseline. If there were differences between the two groups, then the analysis of covariance (ANCOVA) was performed after controlling for the confounding variables. Then, Pearson correlation analysis was used to explore the relationship between BMI and PANSS scores at baseline, and further the relationship between baseline BMI and reductions in PANSS scores after treatment. Further regression analysis was performed to assess the association of BMI at baseline with the decrease in PANSS scores after 12 weeks of treatment after adjusting for various confounding factors.

## 3 Results

The sample included 241 FEMNS patients (128 men, 113 women). Thirty-eight participants were lost before the 2-month follow-up and 53 patients were lost after 2 months of treatment with risperidone. Finally, a total of 91 patients were lost. Table 1 shows the differences between completers and those who dropped out in the follow-up. There were no significant differences in the demographic characteristics and clinical data between completers and drop-outs (all *p* > 0.05).

**TABLE 1:** Demographic characteristics and clinical data.

Variable	Completers (*n* = 150)	Dropouts (*n* = 91)	F or *χ* ^*2*^ (*p*-value)	Low BMI group (*n* = 207)	High BMI group (*n* = 34)	F or *χ* ^*2*^ (*p*-value)
Sex (M/F)	80/70	48/43	0.01 (0.93)	109/98	19/15	0.1 (0.73)
Age (y)	28.1 ± 9.3	26.8 ± 9.0	1.1 (0.31)	27.1 ± 9.3	30.8 ± 8.3	4.8 (0.03)
Weight (kg)	59.6 ± 11.9	57.1 ± 9.8	2.9 (0.09)	56.1 ± 8.3	78.2 ± 10.3	<0.001
BMI (kg/m^2^)	21.6 ± 3.6	20.9 ± 3.0	2.1 (0.15)	20.3 ± 2.1	27.7 ± 2.8	<0.001
Age of onset (y)	26.3 ± 9.2	25.7 ± 9.1	0.3 (0.59)	26.0 ± 9.3	28.2 ± 8.5	1.5 (0.23)
PANSS score						
Positive	21.6 ± 6.3	22.3 ± 6.2	0.8 (0.38)	22.0 ± 6.8	22.8 ± 5.3	0.4 (0.55)
Negative	19.1 ± 6.5	18.3 ± 7.2	0.8 (0.37)	18.9 ± 7.2	18.1 ± 6.2	0.3 (0.58)
General	35.1 ± 10.0	36.1 ± 8.9	0.6 (0.44)	35.7 ± 10.3	37.0 ± 8.9	0.4 (0.52)
Total	75.6 ± 17.7	76.5 ± 16.8	0.1 (0.70)	76.4 ± 18.7	78.0 ± 14.9	0.2 (0.67)

Abbreviations: *y*, years; *ms*, months; *BMI*, body mass index.

After treatment, the mean changes in weight were 2.7 kg (SD = 3.8). According to the criteria of obesity, we identified 34 patients in the high BMI group and 207 patients in the low BMI group. Comparisons of demographic characteristics and clinical data between the high BMI and low BMI groups are shown in Table 1. The mean changes in weight were 0.4 (95% CI: −0.7–1.5) in the high BMI subgroup and 3.1 (95% CI: 2.5–3.6) in the low BMI subgroup. Significant differences were observed in weight gain between the low BMI and high BMI subgroups (*p* < 0.01).

**FIGURE 1:**
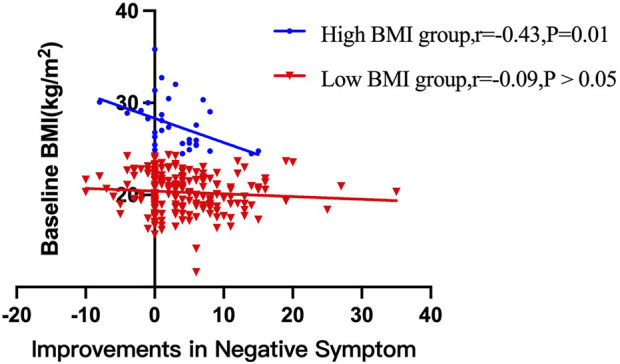
There were significant associations between baseline BMI and improvements in negative symptom improvements after 12 weeks of treatment with risperidone in high BMI group (*p* < 0.05), but not in low BMI group.

After treatment with risperidone, clinical symptoms were significantly improved (Bonferroni corrected all *p* < 0.05) (Table 2). Pearson correlation analysis revealed that baseline BMI was negatively correlated with the improvement in negative symptoms (*r* = −0.14, *p* = 0.03). After subgroup analysis, we found that the negative association was only present in the high BMI group (*r* = −0.43, *p* = 0.01), but not in the low BMI group (Figure 1). Moreover, baseline BMI was not associated with the improvement in positive symptoms, general psychopathology, and PANSS total score (all *p* > 0.05). Further linear regression analysis confirmed that baseline BMI (*β* = −0.15, *t* = −2.2, *p* = 0.027) was a predictive factor for negative symptom improvements, while sex, age and years of education were not associated with negative symptom improvement (all *p* > 0.05).

**TABLE 2:** Reduction of symptoms after 12-week treatment with risperidone in the low BMI and high BMI groups.

	Changes in PANSS and weight (95% CI)^a^	
	High BMI group	Low BMI group
	*n* = 34	*n* = 207
Positive subscore	10.2 (7.8–12.6)**	8.5 (7.6–9.5)**
Negative subscore	2.9 (1.3–4.5)**	4.1 (3.2–4.9)**
General subscore	10.7 (8.2–13.3)**	9.1 (7.7–10.4)**
PANSS total score	28.0 (23.1–32.9)**	21.5 (18.7–24.2)**

^a^ Paired *t*-test to compare the changes of PANSS, and its subscores after treatment with risperidone between baseline and week 12 after treatment. **p < 0.01, *p < 0.05.

Further, among those with low BMI, we found 28 patients were underweight or with undernutrition. To rule out their influence on the results, we reanalyzed the data without these 28 patients and there was no difference in the result (Supplementary Tables S1).

## 4 Discussion

We here found that baseline BMI was associated with weight gain and negative symptom improvements in this relatively large sample of FEMNS patients on 12-week treatment with risperidone. However, we did not find an association between female sex, younger age education levels, and symptom improvements.

This study found that baseline BMI was significantly associated with weight gain after 3 months of risperidone monotherapy. Patients with a lower baseline BMI gained more weight after risperidone medication than those with a higher baseline BMI. Our findings are in line with most previous studies (Jones et al., 2001; Leucht et al., 2013; Huhn et al., 2019) and the recent longitudinal cohort study in FEMN patients with SCZ (Vázquez-Bourgon et al., 2022), providing further evidence for a relationship between baseline BMI/bodyweight and antipsychotic-induced weight gain in SCZ (Gentile, 2009; Verma et al., 2009). Notably, previous evidence-based data reveal that a variety of other risk factors, including ethnicity, young age, recent onset of psychotic symptoms, unemployment, unhealthy lifestyle and low income, may contribute to the rate or magnitude of antipsychotic-induced weight gain during long-term treatments (Gentile, 2009). However, in this study, we did not find an association between weight gain and education years, which was well-known to be strongly associated with unemployment, unhealthy diet and low income. This may be due to the differences between the patients with SCZ we recruited in this study and those in previous studies, considering that a majority of previous studies have investigated weight gain in outpatients on long-term antipsychotic medication.

We further found that baseline BMI was an independent predictor for the improvement of negative symptoms after treatment, after controlling for age, sex, and education years. Indeed, there is evidence to show a close relationship between negative symptoms in SZ patients and BMI at baseline (Jones et al., 2001; Leucht et al., 2013; Sharma et al., 2014; Raben et al., 2017; Huhn et al., 2019), which is consistent with our findings. The findings in the current study were also in line with the studies on FEMNS patients (Zipursky et al., 2005; Venkatasubramanian et al., 2010; Kemp et al., 2013). Importantly, the abovementioned studies have all controlled for confounding factors, such as sex, age, antipsychotic medications, and duration of illness.

However, the exact mechanism remains unclear. It may be due to the shared mechanistic pathway between body weight regulation and negative symptom improvements after antipsychotics. Risperidone has been reported to have a relatively high affinity for D_2_, 5-HT_2_, histamine H_1_ receptors, and NE alpha-2 receptors (Nasrallah, 2008). Animal studies also revealed that obesity induced by a high-fat diet and increased food intake correlated with 5-HT_2_ deficiency (Mercer et al., 1994). In particular, the use of antipsychotics may lead to the preferential metabolism of carbohydrates over fats and further lead to increased fat storage (Tiwari et al., 2018; Kaar et al., 2019). Leptin, a key signal for determining the size of fat depots in the brain, was found to be increased following atypical antipsychotic medication (Brömel et al., 1998; Kraus et al., 1999). On the other hand, dopamine D_2_ receptors play an important role in the reward circuit and in mediating both obesity and therapeutic response to antipsychotics, which may explain the association between BMI and therapeutic benefits in SZ.

Higher BMI was associated with fewer negative symptom changes in this study. It is reported that in patients with higher BMI in the early phase of SZ, brain functional connectivity associated with food cravings and weight control was decreased (Homan et al., 2019). Additionally, the prefrontal cortex of obese patients exhibited altered insulin and DA gene expression, resulting in relatively poorer outcomes than those with lower BMI (Mansur et al., 2018). Altogether, this study suggests that BMI at baseline may be a more sensitive indicator of the therapeutic benefits in the early stage of SZ, as most of the reported correlations between weight gain and therapeutic responses to antipsychotics are mixed.

Some strengths should be mentioned in this study. This is a prospective and longitudinal study examining a well-characterized group of FEMNS patients. Standardized treatment with a single antipsychotic excluded the different impacts of antipsychotic drugs. However, this study has several limitations. First, in this study, obesity-related biomarkers such as glucose, cholesterol, insulin resistance, and lipids were not recorded. Additionally, other metabolic parameters, such as food preference, dietary record, and caloric intake were also not collected. Second, only risperidone was assessed in our study. Therefore, the results of this study may not be generalized to other types of antipsychotics that may be different in metabolic disturbance. Third, the criteria for obesity in our study are only for Chinese, thus the conclusions in this study cannot be generalized to other populations. Fourth, the regulation of body weight may overlap with the pharmacological mechanisms of antipsychotic drugs. Genetic factors are known to be associated with the pharmacodynamics, pharmacokinetics, and adverse effects of antipsychotics. Additional pharmacogenetic analyses may provide new insights into the current findings. Fifth, considering the role of lifestyle, diet, and exercise in explaining the contradictory findings across studies, we did not collect the detailed diet and exercise in the present study.

In conclusion, the current study found that BMI was negatively correlated with the improvements in negative symptoms after treatment with risperidone for 12 weeks in FEMNS patients. In addition, we identified baseline BMI as an independent predictor for negative symptom improvements in SZ. Our study underscores the key role of BMI in the clinical management of patients in the early stage of SZ. These findings provide further evidence that greater efforts should be made to prevent obesity in clinical practice from the early phase of SZ.

## Data Availability

The raw data supporting the conclusion of this article will be made available by the authors, without undue reservation.
